# Impact of hepatitis C virus eradication with direct-acting antivirals on glycidic metabolism

**DOI:** 10.20945/2359-3997000000543

**Published:** 2022-12-01

**Authors:** Jucéli Márcia Hendges Sparvoli, Antonio Cardoso Sparvoli, Samuel de Carvalho Dumith, Afonso Alexandre Pereira, Ana Luisa Machado de Paula, Laís Garcia, Vanusa Belarmino, Vanusa Pousada da Hora, Ana Maria Barral de Martínez, Carla Vitola Gonçalves

**Affiliations:** 1 Universidade Federal do Rio Grande Faculdade de Medicina Rio Grande RS Brasil Faculdade de Medicina, Universidade Federal do Rio Grande (FURG), Rio Grande, RS, Brasil; 2 Universidade Federal do Rio Grande Programa de Pós-graduação em Ciências da Saúde Rio Grande RS Brasil Programa de Pós-graduação em Ciências da Saúde, Universidade Federal do Rio Grande (FURG), Rio Grande, RS, Brasil

**Keywords:** Insulin resistance, direct-acting antivirals, diabetes mellitus, hepatitis C virus, sustained viral response

## Abstract

**Objective:**

To compare the glucose metabolism of patients with chronic hepatitis C virus infection treated with direct-acting antivirals (DAAs) in pretreatment and sustained viral response (SVR) periods.

**Materials and methods:**

This was an intervention pre-post study of 273 patients with chronic hepatitis C virus infection treated with DAAs from March 2018 to December 2019. Glycidic metabolism was evaluated through homeostasis model assessment (HOMA) – insulin resistance (IR) and HOMA-β indices and assessments of insulinemia and HbA1c levels. These parameters were analyzed with a T test by paired comparison of the means of the variables and Wilcoxon's test paired for the median; in the variables with an abnormal distribution, the Z score was generated for the mean in both the pretreatment and SVR periods. Statistical significance was considered at p ≤ 0.05.

**Results:**

Among 273 participants, 125 (45.8%) had prediabetes, and 50 (18.3%) had diabetes. In SVR, there was a significant increase in platelets, albumin, alkaline phosphatase, cholesterol and triglycerides and a significant decrease in aspartate aminotransferase, alanine aminotransferase, gamma GT and bilirubin. The HOMA-IR and HOMA-β indices increased in SVR from 1.95 to 2.29 (p = 0.087) and 71.20 to 82.60 (p = 0.001), respectively. Insulinemia increased from 7.60 μU/mL to 8.90 μU/mL (p = 0.011). HbA1c decreased from 5.6 to 5.4 (p < 0.001). Among patients with prediabetes and those with diabetes, the reduction in HbA1c values was significant (p = 0.006 and p = 0.026, respectively).

**Conclusion:**

SVR significantly impacts and leads to improvement in glucose metabolism in patients with chronic liver disease induced by hepatitis C virus.

## INTRODUCTION

The natural course of chronic hepatitis C virus (HCV) infection is characterized by liver disease and the development of several extrahepatic manifestations that lead to increased morbidity and mortality. The two main metabolic manifestations are insulin resistance (IR) and type 2 diabetes mellitus (T2DM) (1–4). Chronic HCV infection is a risk factor for the development of T2DM, as liver disease progressively worsens (5).

It is estimated that approximately 2/3 of patients may experience extrahepatic manifestations (6–8). The association between HCV and glucose metabolism involves the potential diabetogenic effect of persistent viral infection (9,10). The progression of hepatic fibrosis is considered to be responsible for the development of IR and T2DM; however, changes in glucose metabolism often occur in the early stages of liver disease (2).

The mechanisms by which chronic HCV infection is associated with IR and T2DM involve direct viral effects that interfere with insulin signaling and indirect effects by inducing chronic inflammation through the action of proinflammatory cytokines, chemokines, and other immune-mediated mechanisms (8,11). The HCV genome has also been identified in several tissues other than the liver, such as pancreatic acinar cells and pancreatic duct epithelial cells. This virus appears to be related to pancreatic β-cell dysfunction, but the evidence is scarce (12). Additionally, IR has been shown to be associated with the faster evolution of hepatic fibrosis, steatosis and hepatocellular carcinoma (13).

Some clinical studies suggest improvements in glucose metabolism after antiviral treatment and sustained viral response (SVR) (14,15). SVR is defined as an undetectable viral load at the 12th week after the end of treatment. Most regimens achieve SVR in more than 95% of patients with the use of direct-acting antivirals (DAAs). There is evidence (7) that SVR can reduce mortality from extrahepatic manifestations associated with HCV. SVR by DAAs may also improve the inflammatory state with consequent antiatherosclerotic activity and relevant vascular effects. Therefore, HCV eradication by DAAs allows for a reduction in major adverse cardiac events (MACE) in both the general and prediabetic populations (16,17). Thus, it is plausible to expect that therapies to eradicate HCV, by reducing inflammation, may improve metabolic parameters and reduce the rate of T2DM and IR in patients with chronic HCV infection (14). However, conflicting results regarding the possible metabolic improvement from the use of DAAs emphasize the importance of establishing the effect of SVR on the glucose metabolism of these patients (18). Therefore, this study aimed to compare the glucose metabolism of patients with chronic HCV infection treated with direct-acting antivirals in the pretreatment and SVR periods.

## MATERIALS AND METHODS

This was an intervention pre-post study that evaluated all patients with chronic HCV infection eligible for treatment with DAAs that were treated at the University Hospital Dr. Miguel Riet Correa Jr. at the Federal University of Rio Grande (FURG) from March 2018 to December 2019. The treatment followed the inclusion and duration criteria of the Clinical Protocol and Therapeutic Guidelines for Hepatitis C and Coinfections 2018/2019 (19,20). During this period, 480 patients underwent treatment, of which 151 were excluded, and there were 38 refusals.

The inclusion criteria were age ≥ 18 years, chronic HCV liver disease, compensated cirrhosis (CHILD A) and resident of Rio Grande or São José do Norte. Exclusion criteria were the presence of decompensated cirrhosis (CHILD B and C), coinfection with chronic hepatitis B virus or human immunodeficiency virus (HIV), alcoholic liver disease, severe psychiatric illness, chronic pancreatitis, chronic renal failure, transplant patients, type 1 DM, use of steroids or anabolic steroids, alcohol consumption greater than 50 g/day or not achieving SVR.

A total of 291 patients were selected for this study, of which 13 (4.5%) did not reach SVR and 5 (1.7%) did not undergo examinations or return to consultations, which were considered as lost. Next, the patients completed a precoded standard questionnaire that evaluated sociodemographic and clinical aspects related to HCV and T2DM. After completing the questionnaire, anthropometric measurements of weight, height and waist circumference were performed. Finally, the patients were sent to the laboratory for blood collection.

Weight and height were measured with an adult anthropometric mechanical scale up to 150 kg and with a ruler, respectively, and patients wore light clothing, without accessories and without shoes. Waist circumference (WC) was measured at the midpoint between the last rib and the iliac crest at the end of normal expiration. An abdominal WC measurement of ≤ 93 cm for men and ≤ 79 cm for women was considered adequate, and an abdominal WC measurement of ≥ 94 cm for men and ≥ 80 cm for women was considered inadequate. Body mass index (BMI) was categorized according to the 2016 Brazilian Guidelines for Obesity (21). BMI was calculated using the following formula: BMI = weight/height².

The diagnosis of T2DM, prediabetes and normoglycemia followed the criteria adopted in the Guidelines of the Brazilian Society of Diabetes, 2019-2020 (22). Those patients who had glycated hemoglobin (HbA1c) at values ≥ 5.7 to < 6.4 or fasting glucose ≥ 100 mg/dL to ≤ 125 mg/dL were classified as having prediabetes, and those patients who had HbA1c ≤ 5.6 and/or fasting glucose ≤ 99 mg/dL were classified as normoglycemic. Patients classified as having diabetes had a diagnosis prior to the indication of treatment for HCV.

To assess glucose metabolism, the homeostasis model assessment (HOMA)-IR index (23), HOMA-β index (23), insulin levels, and HbA1c levels were used. The parameters evaluated were measured at the beginning of treatment and at SVR. Below are the formulas used for determining HOMA-IR and HOMA-β:

- HOMA-IR index: fasting glucose x 0.0555 x fasting insulin/22.5;
- HOMA-β index: (20 x fasting insulin)/(fasting glucose x 0.0555) - 3.5.

After data collection, a database was created in the Epi-Data program, where the data were double-typed with an inverse order of entry. Then, comparisons were made between the columns the inconsistencies were adjusted, and the data was subsequently analyzed. For the analysis, the data were exported to the SPSS 20 program, where the sample was first categorized and described. Then, the means of the variables in the pretreatment and in the SVR were compared by the method of paired analysis of the means by the T test, in which p ≤ 0.05 was considered significant. As most variables presented an asymmetric distribution, the Z score of each variable was generated to assess the mean, and the paired Wilcoxon test was used to assess the median and interquartile range (IQR) at the pretreatment and SVR periods. Adjusted analysis was performed for the following variables: demographics (sex, age and skin color), followed by measurement data (BMI and WC) and finally the glycidic profile. We also adjusted for the outcome variables at baseline that had the highest values at baseline and the smallest increases. The insulin outcome was also adjusted for HOMA-IR and HOMA-β. This project was approved by the ethics and research committee in the health area of FURG (CEPAS) under the process number 23116.00516/2018-56.

## RESULTS

Among the 273 study participants, 263 (96.3%) were treatment-naïve, and 10 (3.7%) had already undergone previous treatment (“experienced”). The average age was 57 years, 193 (70.7%) were white, 144 (52.7%) were male and 169 (61.9%) were married or had a partner. Regarding the source of viral infection, 121 patients (54.5%) reported the sharing of syringes as the probable source of contamination, 94 (44.6%) attributed the source of virus to blood transfusion, and 51 (18.7%) did not know how they acquired the virus. Regarding anthropometric data, 200 (73.3%) had inadequate abdominal waist circumference measurements for each sex, and the mean BMI was 27.59. Regarding liver injury, 103 (37.7%) had grade 2 or 3 fibrosis, and 78 (28.6%) had cirrhosis. Regarding the glucose profile, most patients had prediabetes (125, 45.8%) or diabetes (50, 18.3%). In the T2DM group, 8 (16%) used insulin and oral hypoglycemic agents, and 42 (84%) used hypoglycemic agents. For the laboratory results, there was a significant increase in the values of platelets, albumin, alkaline phosphatase, total cholesterol, LDL and triglycerides and a significant decrease in the levels of aspartate aminotransferase (AST), alanine aminotransferase (ALT), gamma-glutamyl transferase and total bilirubin. The HOMA-IR and HOMA-β P indices increased on average after treatment from 1.95 to 2.29 (p = 0.087) and 71.2 to 82.6 (p < 0.001), respectively. The insulin level increased from 7.60 to 8.90 at SVR (p = 0.011). HbA1c was reduced from 5.6 to 5.4 (p < 0.001) (Table 1).

**Table 1:** Anthropometric and laboratory data and comparison parameters in the study of the impact of hepatitis C virus eradication with direct-acting antivirals on glycidic metabolism

		N (%)	Pretreatment	SVR	*p*-value
Age (mean ± SD)		57.03 (±11.11)			
	≤54 years	98 (35.9)			
	55 to 64 years	113 (41.4)			
	≥65 years	62 (22.7)			
Skin Color					
	White	193 (70.7)			
	Not white	80 (29.3)			
Sex					
	Male	144 (52.7)			
	Female	129 (47.3)			
Weight (Mean ± SD)			73.21 (±14.72)	73.54 (±14.69)	0.127
Abdominal WC (Mean ± SD)			94.38 (±11.87)	94.04 (±11.99)	0.206
	Adequate				
	Inadequate	73 (29.3)	27.2 (24.4; 30.3)	27.4 (24.5; 30.8)	0.051*
BMI (median – IIQ)		200 (70.7)			
	≤24.9 (81)	81 (29.7)			
	25 to 29.9 (116)	117 (42.9)			
	≥30 (74)	75 (27.5)	101 (92; 114)	99 (91; 110)	0.727*
Glycemic Profile (median – IIQ)					
	W/normal blood glucose	98 (35.9)			
	W/prediabetes	125 (45.8)			
	W/diabetes	50 (18.3)			
	W/insulin + oral hypoglyc.	8 (17)			
	W/oral hypoglyc.	35 (70)			
	Diet + AF	7 (15)			
Laboratory Results (Mean ± SD)					
	Platelets		195.560 (±64.517)	200.140 (±58.292)	0.039
	Albumin		4.04 (±0.44)	4.35 (±0.32)	<0.001
	AST		63.67 (±51.87)	22.42 (±8.18)	<0.001
	ALT		87.30 (±71.29)	20.59 (±12.14)	<0.001
	Gamma GT		78.39 (±92.93)	30.45 (±31.43)	<0.001
	AF		120.43 (±76.37	167.51 (±87.49)	<0.001
	BT		0.72 (±0.36)	0.66 (±0.36)	0.003
	INR		1.05 (±0.11)	1.04 (±0.13)	0.290
	Total Cholesterol		166.37 (±36.91)	185.52 (±38.41)	<0.001
	LDL		97.30 (±33.86)	111.80 (±36.66)	<0.001
	HDL		49.59 (±14.23)	50.26 (±13.66)	0.652
	Triglycerides		95.44 (±55.67)	104.47 (±67.50)	0.006
Comparison Parameters (median-IIQ)					
	HOMA-IR		1.95 (1.26; 3.06)	2.29 (1.31; 3.19)	0.087*
	HOMA-β		71.2 (49.9; 112.6)	82.6 (53.0; 125.8)	0.001*
	Insulin		7.60 (5.07; 11.2)	8.90 (5.38; 12.6)	0.011*
	HbA1c		5.6 (5.2; 5.9)	5.4 (5.1; 5.8)	<0.001*

* Wilcoxon test for paired.

AF: alkaline phosphatase; ALT: alanine aminotransferase; AST: aspartate aminotransferase; BMI: body mass index; BT: total bilirubin; w/: with; FG: fasting glucose; GT: glutamyltransferase; HbA1c: glycated hemoglobin; HDL: high-density lipoprotein; hypoglycemic; HOMA-β: homeostasis model assessment estimator for β cells; HOMA-IR: homeostasis model assessment estimator for insulin resistance; IIQ: interval interquartile; INR: international normalized ratio; LDL: low-density lipoprotein; N: number; SD: standard deviation; SVR: sustained viral response; WC: waist circumference.

Table 2 shows that insulin increased significantly among patients that were 54 years or younger (8.23 to 9.57; p = 0.001), male (8.90 to 9.69; p = 0.027), and white (9.22 to 10.15; p = 0.006) and in patients that had an inadequate abdominal WC (9.12 to 10.13; p = 0.06), a BMI of 25 to 29.9 (8.28 to 9.54; p = 0.002), prediabetes (9.49 to 11.65; p = 0.001) and diabetes without the use of insulin (8.90 to 10.72; p = 0.048).

**Table 2:** Insulin analysis in relation to demographic, anthropometric and glycidic profile data

		Insulin pre-treatment Mean (SD)	Insulin pre-treatment CI 95%	Insulin SVR Mean (SD)	Insulin SVR CI 95%	*p*-value
Age	
	≤54 years (98)	8.23 (±4.65)	7.33-9.17	9.57 (±4.77)	8.62-10.54	0.001
	55 to 64 years (112)	10.45 (±7.79)	9.13-11.99	10.66 (±7.56)	9.41-12.25	0.599
	≥65 years (61)	8.56 (±4.98)	7.32-9.91	9.17 (±6.36)	7.62-10.88	0.372
Gender						
	Male (144)	8.90 (±6.01)	7.97-9.89	9.69 (±5.71)	8.81-10.58	0.027
	Female (127)	9.59 (±6.51)	8.53-10.96	10.23 (±7.13)	9.11-11.56	0.196
Color						
	White (191)	9.22 (±6.51)	8.31-10.18	10.15 (±6.86)	9.15-11.11	0.006
	Not White (80)	9.23(±5.61)	8.06-10.47	9.45 (±518)	8.27-10.64	0.688
WC abdominal						
	Adequate (76)	8.92 (±5.65)	7.76-10.34	9.08 (±5.72)	7.88-10.46	0.752
	Inadequate (188)	9.12 (±6.31)	8.24-10.06	10.13 (±6.54)	9.22-11.09	0.006
BMI						
	≤ 24.9 (81)	9.00 (±6.07)	7.81-10.36	9.77 (±6.82)	8.44-11.23	0.109
	25 to 29.9 (113)	8.28 (±4.48)	7.53-9.15	9.54 (±5.21)	8.63-10.59	0.002
	≥ 30 (75)	10.94 (±8.24)	9.18-12.94	10.76 (±7.56)	9.22-12.58	0.797
Glycemic Profile						
	Normal G. (96)	8.52 (±4.88)	7.55-9.61	8.29 (±4.63)	7.45-9.31	0.593
	W/Prediabetes (125)	9.49 (±6,71)	8.37-10.75	11.65 (±7.18)	10.46-13.00	0.001
	W/Diabetes (50)	9.89 (±7.29)	8.03-11.91	8.84 (±6.36)	7.24-10.66	0.151
	without insulin (43)	8.90 (±4.52)	7.57-10.21	10.72 (±5.47)	9.14-12.43	0.048
	without insulin (07)	10.32 (±5.74)	6.59-14.44	9.26 (±5.54)	5.52-13.19	0.596

BMI: body mass index; CI: confidence interval; G: glycemia; SVR: sustained viral response; SD: standard deviation; W/: with; WC: waist circumference.

Table 3 shows a significant increase in the HOMA-IR index mean value after SVR among patients who were white (2.43 to 2.73; p = 0.031) and in patients that had a BMI between 25 and 29.9 (2.24 to 2.55; p = 0.012) and an abdominal WC with an inadequate measurement (2.53 to 2.86; p = 0.033).

**Table 3:** Analysis of the HOMA-IR index in relation to demographic, anthropometric and glycidic profile data

		HOMA-IR pre-treatment Mean (±SD)	HOMA-IR pre-treatment CI 95%	HOMA-IR SVR Mean (±SD)	HOMA-IR SVR CI 95%	p-value
Age						
	≤54 years (98)	2.57 (±2.30)	2.11-3.06	2.91 (±3.30)	2.36-3.62	0.112
	55 to 64 years (111)	2.56 (±1.85)	2.20-2.98	2.77 (±1.91)	2.44-3.11	0.246
	≥65 years (62)	2.30 (±1.71)	1.93-2.82	2.38 (±1.90)	1.96-2.94	0.635
Gender						
	Male (144)	2.41 (±1.92)	2.10-2.74	2.57 (±1.99)	2.26-2.93	0.232
	Female (127)	2.61 (±2.07)	2.29-3.01	2.91 (±2.98)	2.48-3.47	0.107
Color						
	White (191)	2.43 (±2.05)	2.14-2.75	2.73 (±2.65)	2.38-3.13	0.031
	Not White (80)	2.69 (±1.84)	2.30-3.10	2.74 (±2.10)	2.31-3.20	0.822
WC abdominal						
	Adequate (79)	2.45 (±1.73)	2.09-2.86	2.42 (±1.84)	2.04-2.86	0.837
	Inadequate (192)	2.53 (±2.10)	2.25-2.86	2.86 (±2.72)	2.49-3.28	0.033
BMI						
	≤24.9 (81)	2.43 (±1.90)	2.04-2.91	2.69 (±2.36)	2.24-3.28	0.171
	25 to 29.9 (116)	2.24 (±1.60)	1.97-2.56	2.55 (±1.66)	2.27-2.87	0.012
	≥30 (74)	3.02 (±2.52)	2.49-3.61	3.06 (±3.54)	2.40-4.03	0.902
	Glycemic Profile					
W/Normal G (98)		2.19 (±2.07)	1.83-2.62	2.37 (±3.02)	1.91-3.11	0.326
	W/Prediabetes (125)	2.40 (±1.71)	2.10-2.71	2.59 (±1.96)	2.25-2.95	0.142
	W/Diabetes (48)	3.44 (±2.27)	2.86-4.10	3.85 (±2.31)	3.24-4.51	0.273
	Without insulin (41)	2.76 (±1.99)	2.21-3.35	2.85 (±1.67)	2.38-3.32	0.675
	With insulin (07)	2.87 (±2.60)	1.36-4.78	3.96 (±2.87)	2.03-5.94	0.410

BMI: body mass index; G: glycemia; CI: confidence interval; G: glycemia; HOMA-IR: Homeostasis Model Assessment Estimative for Insulin Resistance; SD: standard deviation; SVR: sustained viral response; W/: with; WC: waist circumference.

Table 4 shows a significant increase the HOMA-β index in patients that were 55 to 64 years old (79.39 to 95.07; p = 0.026), male (83.22 to 97.80; p = 0.002), and white (78.85 to 100.69; p = 0.035) and in patients that had a BMI between 25 and 29.9 (84.74 for 96.18; p = 0.029), an abdominal WC with an inadequate measurement (87.51 to 100.23; p = 0.012) and prediabetes (84.95 to 93.58; p = 0.041).

**Table 4:** Analysis of the HOMA-β index in relation to demographic, anthropometric and glycidic profile data

		HOMA-β pre-treatment Mean (SD)	HOMA-β pre-treatment CI 95%	HOMA-β SVR Mean (SD)	HOMA-β SVR CI 95%	p-value
Age						
	≤ 54 years (98)	92.90 (±65.05)	80.46-107.55	104.03 (±69.23)	90.12- 119.28	0.107
	55 to 64 years (111)	79.39 (±53.52)	70.30-89.37	95.07 (±76.57)	82.60-110.32	0.026
	≥ 65 years (62)	92.74 (±60.80)	78.99-109.69	94.59 (±56.37)	81.72-108.78	0.815
Gender						
	Male (144)	83.22 (±50.43)	75.34-91.20	97.80 (±55.96)	89.66-106.87	0.002
	Female (127)	91.97 (±68.67)	80.20-103.37	98.65 (±82.66)	85.42-113.47	0.360
Color						
	White (191)	90.88 (±63.20)	82.54-99.86	97.16 (±60.74)	88.37-105.99	0.156
	Not White (80)	78.85 (±49.78)	68.05-89.55	100.69 (±87.67)	83.87-120.55	0.035
WC abdominal						
	Adequate (79)	86.89 (±63.32)	74.29-102.65	93.26 (±62.65)	80.19-107.55	0.301
	Inadequate (192)	87.51 (±58.34)	79.30-95.80	100.23 (±72.36)	90.54-111.27	0.012
BMI						
	≤24.9 (81)	82.53 (±48.94)	72.21-93.90	100.34 (±90.84)	85.26-108.08	0.071
	25 to 29.9 (116)	84.74 (±57.68)	75.07-95.75	96.18 (±61.62)	87.48-110.63	0.029
	≥30 (74)	96.63 (±72.11)	81.12-113.34	99.01 (±53.79)	81.99-123.75	0.779
Glycemic Profile						
	Normal G. (98)	105.92 (±69.69)	92.61-118.78	111.49 (±65.56)	98.22-123.55	0.450
	W/Prediabetes (125)	84.95 (±52.49)	76.64-94.63	93.58 (±53.46)	83.36-103.20	0.041
	W/Diabetes (48)	55.55 (±37.80)	45.42-66.45	83.10 (±103.82)	58.34-115.43	0.077
	without insulin (41)	99.36 (±82.42)	75.32-126.34	106.15 (±61.24)	88.04-125,15	0.521
	without insulin (07)	65.20 (±52.04)	30.53-102.78	149.67 (±230.8)	39.41-324.93	0.411

BMI: body mass index; G: glycemia; HOMA-β: Homeostasis Model Assessment for β cell; W/: with; WC: waist circumference.

Table 5 shows a significant decrease in HbA1c levels after SVR among patients that were 65 years old or older (5.66 to 5.44; p = 0.019), male (5.63 to 5.48; p = 0.012), and white (5.66 to 5.52; p = 0.010) and in patients that had abdominal WC with an inadequate measurement (5.87 to 5.60; p = 0.019), a BMI greater than or equal to 30 (5.77 to 5.47; p = 0.006), diabetes (7.12 to 6.67; p = 0.029) and prediabetes (5.55 to 5.40; p = 0.006).

**Table 5:** Analysis of HbA1c in relation to demographic, anthropometric, and glycemic profile data

		HbA1c pre-treatment Mean (SD)	HbA1c pre-treatment CI 95%	HbA1c SVR Mean (SD)	HbA1c SVR CI 95%	p-value
Age						
	≤54 years (98)	5.63 (±0.72)	5.49-5.77	5.54 (±0.75)	5.40-5.70	0.198
	55 to 64 years (113)	5.86 (±1.18)	5.66-6.09	5.72 (±1.16)	5.53-5.93	0.119
	≥65 years (61)	5.66 (±0.87)	5.48-5.90	5.44 (±0.66)	5.28-5.61	0.019
Gender						
	Male (144)	5.63 (±0.73)	5.51-5.74	5.48 (±0.83)	5.35-5.62	0.012
	Female (129)	5.85 (±1.17)	5.66-6.06	5.71 (±1.02)	5.55-5.89	0.079
Color						
	White (193)	5.66 (±0.84)	5.55-5.79	5.52 (±0.78)	5.42-5.64	0.010
	Not white (80)	5.90 (±1.21)	5.67-6.16	5.76 (±1.21)	5.52-6.04	0.196
WC						
	Adequate (80)	5.87 (±1.15)	5.63-6.12	5.60 (±1.07)	5.37-5.86	0.019
	Inadequate (193)	5.67 (±0.88)	5.55-5.81	5.59 (±0.87)	5.47-5.71	0.087
BMI						
	≤24.9 (81)	5.77 (±1.06)	5.52-5.97	5.71 (±1.20)	5.48-5.99	0.918
	25 to 29.9 (117)	5.72 (±0.91)	5.56-5.89	5.59 (±0.87)	5.44-5.76	0.090
	≥30 (75)	5.77 (±0.97)	5.58-6.02	5.47 (±0.64)	5.33-5.62	0.006
Glycemic Profile						
	Normal G. (98)	5.26 (±0.34)	5.19-5.33	5.29 (±0.52)	5.19-5.40	0.549
	W/Prediabetes (125)	5.55 (±0.38)	5.48-5.61	5.40 (±0.51)	5.31-5.50	0.006
	W/Diabetes (50) without	7.12 (±1.46)	6.75-7.50	6.67 (±1.48)	6.31-7.07	0.026
	insulin (43)	6.56 (±1.31)	6.19-6.99	6.39 (±1.59)	5.96-6.88	0.435
	without insulin (07)	7.34 (±2.07)	6.12-8.94	6.40 (±1.42)	5.47-7.47	0.102

BMI: body mass index; CI: confidence interval; G: glycemia; HbA1c: glycated hemoglobin; SD: standard deviation; SVR: sustained viral response, W/: with; WC: waist circumference.

After the adjusted analysis, we observed that only age (p = 0.05) and skin color (p = 0.01) remained associated with insulin in SVR. For the HOMA-IR index, the skin color variable (p = 0.01) remained significant, and for the HOMA-β index, patients with prediabetes showed a statistical significance of p = 0.02. For the variable HbA1c, BMI (p = 0.03) and prediabetes (p < 0.01) were significantly associated with the decrease in this parameter in SVR.

## DISCUSSION

In the present study, most patients had prediabetes (45.8%) or T2DM (18.3%), representing a total of 64.1% of the sample population, which confirms the important association between HCV and alterations in glucose metabolism (2,3). This finding corroborates the results of other authors (24,25) indicating that HCV is a diabetogenic agent. Besides, the reduction of cardiovascular and cardiometabolic risk is the great challenge of the future. Following SVR via DAAs, both a significant reduction in MACE in general and prediabetic population, and a reduction in progression towards diabetes (26) have recently been observed. In addition to an important extra-hepatic impact, SVR by DAAs leads to an important reduction in the risk of hepatocellular carcinoma (17,26,27). These results underline the importance of sustained clearance of HCV by DAAs on the overall clinical outcome of patients.

This perception increases the interest in viral eradication in these individuals. In particular, we analyzed the effect of SVR with DAAs, which induce eradication in 95% of patients (3,28). In addition, interferon treatment causes most patients to lose a significant amount of weight, which makes it difficult to interpret metabolic assessment results (4). Thus, the maintenance of these anthropometric parameters at the end of treatment with DAAs allows us to establish more clearly the possible effect of viral eradication on the glycidic metabolism of the patients studied. In this study, there were no changes in mean weight, BMI and abdominal WC between the pretreatment and SVR periods, and therefore these parameters, which are relevant to glucose metabolism, did not affect the analyses performed.

Regarding the evaluation of glucose metabolism, it was observed that there was a significant increase in the HOMA-IR index, suggesting that insulin resistance did not improve. However, there was an improvement in β cell function, demonstrated by the significant improvement in the HOMA-β index, which was associated with increased insulin and decreased HbA1c levels. The results of the multivariate analysis corroborated this understanding. Thus, the present study suggests an important impact of SVR on glucose metabolism in patients with chronic HCV infection when they were treated with DAAs. There is no consensus in the literature on the effect of SVR on glycidic metabolism. Li and cols. (29) reported that SVR does not improve glycemic control in patients with T2DM, while other authors (24,25,30) reported improvements in insulin sensitivity and glycemic control. A retrospective study with 281 participants (31) identified a significant improvement in fasting glucose and a reduction in the rate of prediabetes after SVR, but this beneficial effect did not extend to patients with cirrhosis. In contrast, a Brazilian study (32) with 150 patients demonstrated that insulin resistance did not improve after SVR was obtained with DAAs. In the same study, the authors found that after excluding patients with DM and those with a normal baseline HOMA-IR index (<2.5), the mean blood glucose level, insulin level and HOMA-IR index decreased significantly (p = 0.02) after SVR. Therefore, data on the activity of SVR on glycidic metabolism remain inadequate and show conflicting divergences and results (33).

Regarding anthropometric parameters, most patients were overweight (42.9%) or obese (27.5%), and 73.3% had a waist circumference with an inadequate measurement for their sex. When the HOMA-IR of these groups was evaluated, it was found that there was no improvement after SVR. In contrast, the study by Russo and cols. (4), which studied 138 patients without diabetes, showed that only 2.2% of the patients were obese and demonstrated a significant improvement in IR in SVR, but the results emphasized that the higher the BMI was, the lower the probability of improvement in IR. It is suggested that the significant percentage of overweight and obese patients in the present study contributed to the difficulty in achieving improvement in IR during SVR, as assessed by the HOMA-IR index.

Despite the lack of improvement in HOMA-IR, in all subgroups analyzed, there was a generalized tendency of HOMA-β to approach the ideal value of 100%, in addition to the statistically significant improvement in patients who had an inadequate abdominal WC, were overweight or had prediabetes. Similarly, insulin levels also increased in those who had an inadequate abdominal WC, were overweight, had prediabetes, and had T2DM without insulin use. The improvement of parameters related to β cell function (HOMA-β, insulinemia) in several subgroups raises the consideration of the possibility of another role of HCV in glycidic metabolism. An experimental work (34) indicated that, in addition to IR, pancreatic β dysfunction is central in the progression of DM. The researchers demonstrated that HCV can directly induce the death of β cells, confirming a direct cytopathic effect on pancreatic islets, which was reinforced by the fact that the HCV genome was found in pancreatic cells. Additionally, a study by Shehab-Eldin and cols. (18) found that SVR with DAAs improves glycemic control, with a reduction in fasting glucose at the end of treatment. The authors explained that this finding reflected an improvement in β cell function, as HCV could directly inhibit insulin secretion, which would improve after virus eradication. The study by Huang and cols. (24), with 65 patients, also did not see improvement in IR at the end of treatment but did find a significant improvement in β cell function when SVR was achieved. Thus, the data obtained in the present study suggests a greater influence of HCV on pancreatic tissue than on peripheral IR, which involves tissues less affected by HCV, such as skeletal muscles and adipose tissue.

A practical aspect that was verified in this study was the improvement in HbA1c levels, an important parameter in diagnostic and therapeutic decisions in the clinical routine. There was a significant improvement in HbA1c levels in obese patients with prediabetes and T2DM. The literature shows studies demonstrating improvement in IR in SVR, but the results on glycemic control are still unclear. A study by Ciancio and cols. (15) with 110 patients with T2DM treated with DAAs demonstrated a significant reduction in HbA1c levels after treatment. Similarly, a study conducted with 240 patients (35) showed improvement in HbA1c levels in SVR, both in people with and without T2DM. Huang and cols. (24), when studying the effects of SVR on HbA1c levels in 65 patients, found no improvement. The present study suggested a better glycemic control in these patients after SVR.

Despite the significant improvement in several of the glucose metabolic parameters analyzed, it was verified that this beneficial effect does not occur in all patients. This suggests that HCV is not the only factor involved in the pathogenesis of T2DM in patients with chronic liver disease. Other factors can directly affect glycemic control (11): the specific genetic profiles of patients, disorders in liver function and exacerbated inflammatory response that may persist after eradication of HCV in some patients with established liver disease (25). The early recognition of the systemic effects of chronic HCV infection, especially IR and T2DM, increases the importance of universal treatment of these patients, avoiding additional and potentially permanent deleterious consequences, both hepatic and extrahepatic (3,29), in addition to improving health-related quality of life aspects (10). Thus, the present study suggests that SVR influences glycidic metabolism, with improvement in β cell function and facilitation of better glycemic control, as evaluated by HbA1c levels.

The limitations of the present study include a relatively short follow-up; a longer evaluation could provide additional information about the effect of SVR on glucose metabolism and whether any improvements in glycemic control would remain long-term. The possible effect of hepatic steatosis on glycemic control during SVR has not been studied. Despite the above limitations, we suggest that even if there is no improvement in IR upon SVR, the improvement in the activity of β cells, evaluated here with the increase in HOMA-β and insulin in patients with obesity, prediabetes and diabetes, should be better studied. Perhaps further experimental studies evaluating the cytopathic effect of the virus at the cellular level can establish the route of association of HCV with pancreatic cells.
